# Hospitalization and Emergency Department Costs in Patiromer and Sodium Zirconium Cyclosilicate Users

**DOI:** 10.34067/KID.0000001195

**Published:** 2026-04-01

**Authors:** Nihar R. Desai, Charuhas Thakar, Tejas Desai, Marco Soro, Jeffrey Budden, Nathan Kleinman

**Affiliations:** 1 Yale School of Medicine, New Haven, Connecticut; 2 Wellcome-Wolfson Institute for Experimental Medicine, Queen's University Belfast, Belfast, United Kingdom; 3 CSL Vifor US, King of Prussia, Pennsylvania; 4 CSL Vifor Glattbrugg, ZH, Switzerland; 5 Kleinman Analytic Solutions, LLC, Paso Robles, California

**Keywords:** CKD, heart failure, hospitalization, renin-angiotensin system, sodium (Na+) transport, hyperkalemia

## Abstract

**Key Points:**

- This study shows a statistically significant 15% potential heart failure hospitalization and edema event cost savings among patiromer users.
- Annual costs were $52,153 in the sodium zirconium cyclosilicate cohort and $44,245 in the patiromer cohort, an annual difference of $7908 per person.
- In addition to clinical benefits, these results highlight financial implications for health plans, value-based care organizations, and patients

**Background:**

A previous study using US electronic health records directly compared the risk of heart failure–related or edema-related hospitalizations and edema-related emergency department (ED) visits for hyperkalemic patients using patiromer or sodium zirconium cyclosilicate. Incidence rates for all three clinical outcomes favored patiromer, but the previous study did not capture costs.

**Methods:**

The current investigation combines the patiromer and sodium zirconium cyclosilicate cohort heart failure hospitalization and edema event rates from the previous study with mean event and prescription costs from 2019 to 2021 Optum Clinformatics data. The resulting heart failure–related and edema-related event and medication costs were then compared between patiromer and sodium zirconium cyclosilicate cohorts. We inflation-adjusted those to 2024 US dollars.

**Results:**

For patiromer, costs for heart failure hospitalizations, edema hospitalizations, and edema ED visits were $30,269.05, $2520.13, and $31.67 per person-year, respectively. For sodium zirconium cyclosilicate, costs for heart failure hospitalizations, edema hospitalizations, and edema ED visits were $39,478.29, $4061.48, and $51.04, respectively. Medication costs per person-year were $11,423.68 and $8561.71 for patiromer and sodium zirconium cyclosilicate, respectively. The aggregated cost savings with patiromer was $7907.99 per person-year (95% confidence interval, $5453.58 to $10,362.40) for all heart failure hospitalization and edema hospitalization and ED events, adjusted for both inflation and medication costs.

**Conclusions:**

Total event and medication costs were $44,245 in the patiromer cohort and $52,153 in the sodium zirconium cyclosilicate cohort. The $7908 difference represents a 15% (95% confidence interval, 10% to 20%) cost savings associated with patiromer from all events, including medication costs and adjusted for inflation.

## Visual Abstract

**Figure:**
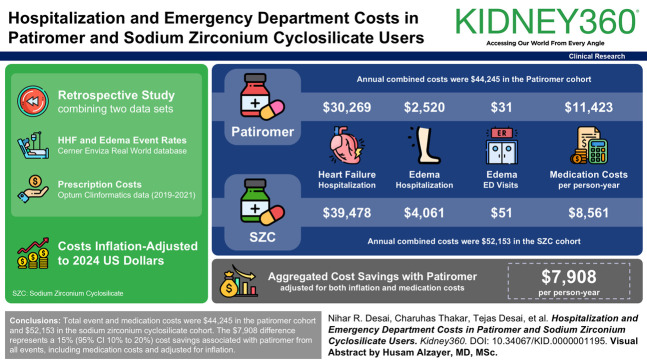

## Introduction

Hyperkalemia (HK) is a common complication in patients with CKD, diabetes, and heart failure. This risk can be higher when renin-angiotensin-aldosterone synthesis inhibitors that are guideline-recommended to reduce the risk of morbidity and mortality of patients with kidney disease are used.^1^ Kidney Disease Improving Global Outcomes 2022 Clinical Practice Guidelines recommend treating HK with a low potassium diet or agents such as potassium binders and diuretics rather than discontinuing renin-angiotensin-aldosterone synthesis inhibitors in the presence of HK.^2^ Management of volume status and its consequences is also critical in this patient population, and patients with CKD are recommended to limit their sodium intake to avoid these consequences.^2^

Potassium binder medications sodium zirconium cyclosilicate and patiromer are effective in treating hyperkalemia and maintaining normokalemia in patients with CKD and/or heart failure. Patiromer exchanges calcium for potassium, whereas sodium zirconium cyclosilicate exchanges sodium and hydrogen for potassium.

A study by Desai *et al*. published in 2024 retrospectively assessed the occurrence rates of potential electrolyte and fluid imbalance-related encounters including hospitalizations for heart failure and hospitalizations and/or emergency department (ED) visits for edema. Desai *et al*. compared these encounters in nearly 30,000 new users of patiromer or sodium zirconium cyclosilicate. Hospitalizations for heart failure were 0.093 per person per month (PPPM) in the sodium zirconium cyclosilicate cohort and 0.071 PPPM in the matched patiromer cohort. Similarly, rates of edema events were 0.015 versus 0.009 PPPM in sodium zirconium cyclosilicate versus patiromer, respectively. The study concluded that sodium zirconium cyclosilicate users were associated with a significantly higher risk of these prespecified events than users of patiromer.^3^

We hypothesized the event rate differences reported in the study by Desai *et al*. would result in a significant cost difference. This study estimates the cost differences for hospitalizations and/or ED visits between patients using patiromer versus sodium zirconium cyclosilicate, using the events rates from Desai *et al*. and event costs from claims. Moreover, this study includes medication costs for both cohorts derived from a similar claims dataset to estimate total event and drug costs.

## Methods

The heart failure hospitalization and edema event rates used in this study were reported in Desai *et al*.^3^ and came from the Cerner Enviza Real-World database. These data are from electronic health records (EHR), given with the consent of clinics and hospitals, and include deidentified data of more than 100 million patients in the United States with information about patient demographic, pharmacy, clinical, outpatient, and admission data.

Desai *et al*. measured outcomes of hospitalizations for heart failure (HHF) using any diagnosis position (HHF-any), HHF using only admission discharge diagnoses (HHF-primary), deaths, and severe edema events (hospitalizations and ED visits) for patients with and without a history of heart failure. The study by Desai *et al*. used Systemized Nomenclature of Medicine and as well as International Classification of Diseases (ICD) diagnosis codes to identify outcomes for heart failure and edema.^3^

We used a licensed extract from Optum's deidentified Clinformatics Data Mart (CDM) covering calendar years 2019–2021 (inclusive) to obtain mean cost information for heart failure and edema hospitalizations, edema ED visits, and 30-count prescriptions of both patiromer and sodium zirconium cyclosilicate. CDM holds inpatient, outpatient, prescription, and professional service medical claims for more than 75 million individuals with commercial or Medicare Advantage health insurance plans in the United States.

This study used ICD, Tenth Revision (ICD-10) codes with the first three digits of I50 in any position in the claim to determine heart failure hospitalizations. This study identified edema hospitalizations and ED visits using ICD-10 codes beginning with R60 in any position. Methods and a thorough description of the analysis are detailed elsewhere.^4,5^

Hospitalization and ED visit costs were inflation-adjusted to 2024 dollars using the hospital services consumer price index. Drug costs were inflation-adjusted to 2024 dollars using the prescription drug consumer price index.

### Statistical Analysis

We converted HHF and edema event rates, rate differences between the patiromer and sodium zirconium cyclosilicate cohorts, and 95% confidence intervals (CI) for the rate differences from the Desai study from PPPM rates to per 100 person-year rates by multiplying by 1200 (100 times 12 months) to yield the annual rate scaled to 100 patients. We multiplied costs per heart failure and edema hospitalization and costs per edema ED visit from the CDM by the Desai *et al*. HHF and edema hospitalization and ED rates, rate differences, and rate difference CIs for the patiromer and sodium zirconium cyclosilicate cohorts to obtain per 100 person-year event costs, cost differences, and cost CIs. Next, we multiplied costs per 30-count prescriptions of patiromer or sodium zirconium cyclosilicate from the CDM in the same way to obtain per 100 person-year medication costs. We then added the per 100 person-year event costs and medication costs together and compared them between cohorts. We performed statistical analysis using Microsoft SQL Server Management Studio and Microsoft Excel.

## Results

This study incorporated 2,095,607 heart failure hospitalizations with an average cost of $35,412.03 per hospitalization, 514,859 edema hospitalizations averaging $42,754.10 per hospitalization, and 441,342 edema ED visits with an average cost of $626.80 per ED visit. Edema hospitalizations accounted for 54% of all edema events. The average cost of the 34,578 patiromer prescriptions (quantity=30/mo) was $951.97, in contrast to $713.48 for the 13,181 sodium zirconium cyclosilicate prescriptions (quantity=30/mo).

Table 1 presents the number and average cost of hospital events and prescriptions per 100 person-years.

**Table 1:** Heart failure and edema event cost savings model

Model Input: Events^a^	*N*	Mean Cost, $	Model Input: Prescription Fills (30 count)^a^	*N*	Mean Cost, $
HHF	2,095,607	35,412.03			
Edema hospitalizations	514,859	42,754.10	Patiromer	34,578	951.97
Edema ED visits	441,342	626.80	SCZ	13,181	713.48
	**Patiromer**	**SCZ**	**Savings with Patiromer**	**95% CI**	
**Model inputs/100 person-yr** ^b^					
HHF event rate	85.5	111.5	26.0	20.4 to 31.6	
Edema event rate^c^	10.9	17.6	6.7	4.6 to 8.8	
	**Patiromer**	**SCZ**	**Savings with Patiromer**	**95% CI**	
**Model outputs/100 person-yr**					
HHF/edema hospitalizations and ED cost, $	3,282,085	4,359,081	1,076,996	831,555 to 1,322,437	
K^+^ binder cost, $^d^	1,142,368	856,171	−286,197		
**Total cost, $**	4,424,452	5,215,252	790,799	545,358 to 1,036,240	

$, inflation-adjusted to 2024 US dollars; CI, confidence interval; ED, emergency department; HHF, hospitalization for heart failure; K^+^, potassium; SCZ, sodium zirconium cyclosilicate.

a Optum Clinformatics 2019–2021.

b Desai *et al*.^3^

c 54% hospitalizations and 46% emergency department visits.

d K^+^ binder cost calculation assumes continuous medication coverage.

Our analysis found $3,282,085 in costs per 100 person years for HHF and edema events with patiromer in comparison with $4,359,081 in costs with sodium zirconium cyclosilicate. While patiromer medication costs ($1,142,368) were greater than those of sodium zirconium cyclosilicate ($856,171), total event and medication costs in the patiromer cohort were $790,799 (95% CI of $545,358 to $1,036,240) less than in the sodium zirconium cyclosilicate cohort. Table 2 presents the potential cost offsets for various numbers of person-years.

**Table 2:** Cost offsets for various numbers of person-years

	Patiromer Total Cost, $	Sodium Zirconium Cyclosilicate Total Cost, $	Potential Cost Offset with Patiromer, $^a^	95% CI, $
Per person-yr (annual)	44,245	52,153	7908	5454 to 10,362
Per 5 person-yr	221,223	260,763	39,540	27,268 to 51,812
Per 25 person-yr	1,106,113	1,303,813	197,700	136,340 to 259,060
Per 50 person-yr	2,212,226	2,607,626	395,400	272,679 to 518,120
Per 100 person-yr	4,424,452	5,215,252	790,799	545,358 to 1,036,240

$, inflation-adjusted to 2024 US dollars; CI, confidence interval.

**Figure 1:**
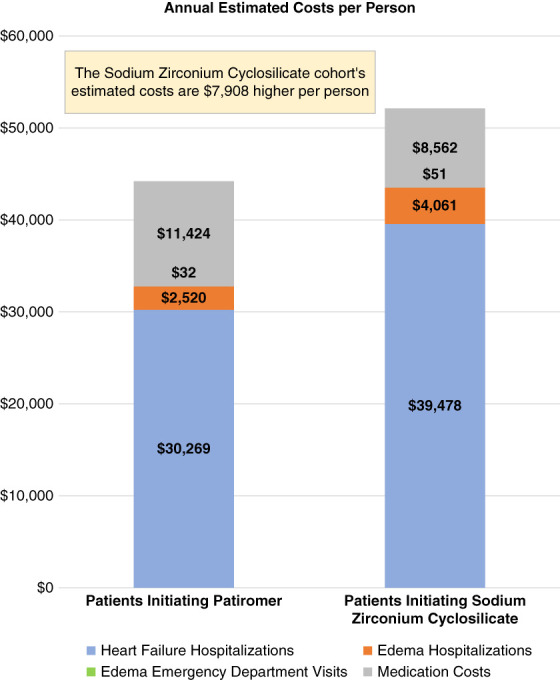
Annual estimated costs per person.

Figure 1 shows the results of this study on an annual per-person basis. For patiromer, costs for heart failure hospitalizations, edema hospitalizations, and edema ED visits were $30,269.05, $2520.13, and $31.67 per person-year, respectively. For sodium zirconium cyclosilicate, costs for heart failure hospitalizations, edema hospitalizations, and edema ED visits were $39,478.29, $4061.48, and $51.04, respectively. Medication costs per person-year were $11,423.68 and $8561.71 for patiromer and sodium zirconium cyclosilicate, respectively. On a PPPM basis, HHF and edema events totaled $2735.07 and $3632.57 for patiromer and sodium zirconium cyclosilicate, respectively. Adding in the monthly medication costs stated above, total monthly costs were $3687.04 and $4346.04, respectively, yielding a monthly savings of $659.00 for patiromer. The cost savings for patiromer represent a 15% decrease relative to sodium zirconium cyclosilicate ($659.00/$4346.04).

## Discussion

Although both patiromer and sodium zirconium cyclosilicate are not indicated for the prevention of the described events, the current analysis applied HHF and edema hospitalization and ED event rates from a propensity-matched retrospective cohort study^3^ to average HHF and edema event costs and medication costs from claims data and found that combined event and medication costs were significantly lower in patients taking patiromer compared with those taking sodium zirconium cyclosilicate. The combined cost was lower in the patiromer cohort by $790,799 per 100 person-years or $7908 per person per year (15%, 95% CI, 10% to 20%).

Using a similar methodology, Kleinman *et al*.^4^ applied claims-based mean event costs to HHF and edema event rates in patiromer and sodium zirconium cyclosilicate users from an earlier claims data study by Zhuo *et al*.^5^ and obtained similar cost comparisons. Using claims data to measure event rates has the benefit of a broad scope, as claims data capture services from all providers caring for a patient and all locations of care, but claims data are limited in the amount of detail available (laboratory values, procedure outcomes, *etc*.). The study by Zhuo *et al*. found a statistically significant rate difference in hospitalizations for heart failure (from any diagnosis code position) between patiromer patients and sodium zirconium cyclosilicate patients (10.7 hospitalizations per 100 person-years [95% CI, 2.6 to 18.8]; lower rates for patiromer); however, the hazard ratio was not statistically significant. In addition, Zhuo *et al*. found a statistically significant rate difference for edema hospitalizations and ED visits (any position) between patiromer and sodium zirconium cyclosilicate patients (3.6 events per 100 person-years [95% CI, 1.7 to 7.1]; lower rates for patiromer). The hazard ratio was statistically significant. Kleinman *et al*. converted these rates into costs and added them to medication costs. The resulting combined cost difference found by Kleinman *et al*. was $1428 per person per year (lower for patiromer) but was not statistically significant (95% CI, -$1508 to $4652).

**Figure 2:**
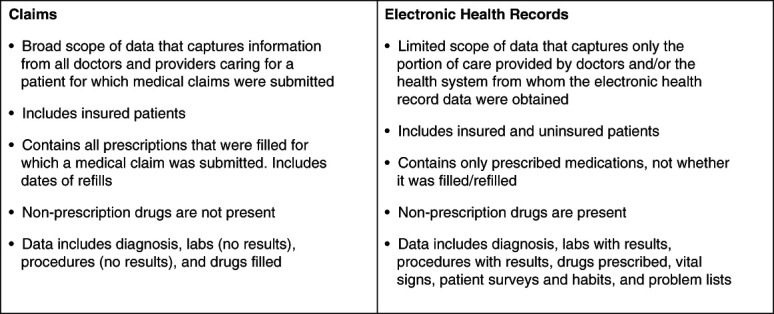
**Claims versus EHR.** EHR, electronic health records.

Conversely, Desai *et al*.'s^3^ use of EHR data to measure event rates has the benefit of procedure and lab details, but it may not capture services from all providers and all locations of care (see Figure 2). The differences in cost and savings estimates between this study and the study by Kleinman *et al*. are due to the differences in event rates between the studies by Zhuo *et al*. and Desai *et al*. (Desai's event rates were two to three times higher than Zhuo's).^3,5^ The Zhuo and Desai studies applied similar methodologies to different data sources (Optum Clinformatics claims data for the former versus Cerner EHR data for the latter), but rate differences may also be due to Desai's shorter follow-up period (median follow-up in the Zhuo study was 58 days, but the median follow-up in Desai's study was 35–48 days).^3^ By contrast, Desai *et al*. studied more patients (9929 patiromer users and 19,849 sodium zirconium cyclosilicate users) than Zhou *et al*. studied (2839 patiromer users and 1126 sodium zirconium cyclosilicate users).

Ramirez *et al*.^6^ developed a cost analysis model based on the HHF and edema event rates in the Zhuo *et al*. study with cost data from Spain and the United Kingdom that resulted in data showing a 2022 cost savings of €107 and £630 per patient per year of treatment, respectively. The analysis also found at a population level the annual cost savings were €30.6 million in Spain and £801.7 million in the United Kingdom for those that used patiromer versus sodium zirconium cyclosilicate.

This study has several strengths. This study is the largest and most contemporary of only three studies that have compared hospitalization and ED costs between patiromer and sodium zirconium cyclosilicate patients.^4,6^ In addition, the cost differences found in this study are based on HHF and edema event rates from EHR data in the Cerner Enviza Real-World database, and they yield a conclusion congruent with the conclusion from Kleinman *et al*.,^4^ whose cost differences were based on HHF and edema event rates from Optum's Clinformatics claims data. See Figure 2 for details differentiating claims and EHR data sources. Combined use of claims data with EHR data may give a more thorough, clear, and complete picture of the study question.

In addition, confounding by indication between the cohorts is likely not an issue in this study for several reasons. First, the Desai *et al*. heart failure and edema event rates were calculated in a propensity score–matched population where sodium zirconium cyclosilicate (SZC) users were matched to patiromer users on history of heart failure, stages 3–5 CKD, diabetic nephropathy, use of heart failure-relevant medications, and demographics. Second, use of patiromer and SZC in our study was assumed to be equal. Finally, previous studies of patiromer and SZC users show either comparable number or severity of comorbid conditions between patiromer and SZC users^7,8^ or more severe comorbid conditions in patients exposed to patiromer than SZC.^9^ Thus, the SZC population's higher heart failure and edema costs are likely not because it is a more severely ill population than the patiromer population.

This study has limitations. The study applies average costs to event rates from a previous study rather than using costs and rates from the same dataset. There may be differences in the patients or setting used by Desai *et al*. to calculate event rates and the patients or setting found in Optum's claims data that we used to calculate event costs. Owing to geographic cost variation, there may be discrepancies between the national cost values reported here and those within specific states or regions.

Medication costs were not dose-specific nor were event rates dose-adjusted. Sodium zirconium cyclosilicate patients taking 15 g would need to use two packages, which may increase sodium zirconium cyclosilicate medication cost and our analysis's reported possible cost offset. However, we attempted to use costs for any dose for a 30-day period and then annualized the costs. Annualizing the medication costs assumes all patients in both cohorts used the drugs continuously for 12 months, which may not represent real-world treatment duration or dosing. In addition, these claims data did not capture potassium, sodium, or other laboratory values. This study was based on a propensity-matched analysis versus a randomized controlled trial. However, recent randomized, placebo-controlled trials have shown signals for a higher rate of adverse events related to heart failure with sodium zirconium cyclosilicate^10,11^ and an earlier study showed higher rates of edema.^12^

Finally, the use of sodium zirconium cyclosilicate in sodium-sensitive patients or patients at risk for volume overload may require additional imaging modalities or laboratory diagnostics to assess volume.^13–16^ These assessments have costs beyond those of a physical exam, including facility fees, operator costs, and interpretation costs by additional specialist physicians. These costs were not captured in this study but would be added to sodium zirconium cyclosilicate use in accordance with full absorption costing and compliance with generally accepted accounting principles.^17^

This study shows a statistically significant 15% potential cost savings for heart failure hospitalizations, edema hospitalizations and EDs visits, and medications associated with patients taking patiromer compared with patients taking sodium zirconium cyclosilicate. Annual combined costs were $52,152.52 in the sodium zirconium cyclosilicate cohort and $44,244.52 in the patiromer cohort, yielding a difference of $7907.99 per person per year. In addition to clinical benefits, these results highlight financial implications for health plans, hospital systems, value-based care organizations, federal agencies, and patients. Stakeholders should consider the current analysis and related studies^3–6^ in health care planning.

## Data Availability

Data belong to a third party, and authors are not authorized to share the data. Identity of Third Party: Optum. Reason for Restriction: Optum data were used to calculate hospitalization and emergency department cost averages and medication cost averages. Access to the Optum data is no longer available.
